# Predictive value of postoperative C-reactive protein-to-albumin ratio in anastomotic leakage after esophagectomy

**DOI:** 10.1186/s13019-021-01515-w

**Published:** 2021-05-17

**Authors:** Chi Zhang, Xiao Kun Li, Li Wen Hu, Chao Zheng, Zhuang Zhuang Cong, Yang Xu, Jing Luo, Gao Ming Wang, Wen Feng Gu, Kai Xie, Chao Luo, Yi Shen

**Affiliations:** 1 Department of Cardiothoracic Surgery, Jinling Hospital, Medical School of Nanjing University, Nanjing, China; 2 Department of Cardiothoracic Surgery, Jinling Hospital, School of Medicine, Southeast University, Nanjing, China; 3 Department of Cardiothoracic Surgery, Jinling Hospital, School of Clinical Medicine, Nanjing Medical University, Nanjing, China; 4 Department of Thoracic Surgery, Xuzhou Central Hospital, Xuzhou School of Clinical Medicine of Nanjing Medical University, Nanjing, China; 5 Department of Cardiothoracic Surgery, Jinling Hospital, School of Clinical Medicine, Southern Medical University, Guangzhou, China

**Keywords:** C-reactive protein, Albumin, Anastomotic leakage, Esophagectomy, Risk factor

## Abstract

**Introduction:**

Among the many possible postoperative complications, anastomotic leakage (AL) is the most common and serious. Therefore, the purpose of this study was to explore the ability of various inflammatory and nutritional markers to predict postoperative AL in patients after esophagectomy.

**Methods:**

A total of 273 patients were retrospectively evaluated and enrolled into this study. Perioperative, surgery-related, tumor-related and laboratory tests data were extracted and analyzed. The discriminatory ability and optimal cut-off value was evaluated according to the receiver operating characteristic (ROC) curve analysis. Univariate and multivariate analyses were performed to access the potential risk factors for AL.

**Results:**

The overall incidence of AL was 12.5% (34/273). C-reactive protein-to-albumin ratio (CRP/ALB ratio) [AUC 0.943 (95% confidence interval (CI) = 0.911–0.976, *p* <  0.001)] and operation time [AUC 0.747 (95% CI = 0.679–0.815, *p* <  0.001)] had the greatest discrimination on AL prediction. Multivariate analysis demonstrated that CRP/ALB ratio and operation time were two independent risk factors for AL, and CRP/ALB ratio (OR = 102.909, *p* <  0.001) had an advantage over operation time (OR = 9.363, *p* = 0.020; Table 3).

**Conclusion:**

Operation time and postoperative CRP/ALB ratio were two independent predictive indexes for AL. Postoperative CRP/ALB ratio greater than 3.00 indicated a high risk of AL. For patients with abnormal postoperative CRP/ALB ratio, early non-operative treatment or surgical intervention are needed to reduce the serious sequelae of AL.

## Introduction

Esophageal cancer (EC) is the eighth most common cancer and the sixth leading cause of cancer-related death worldwide [1]. In addition to radiotherapy, chemotherapy, and molecular targeted therapy, surgery is still considered to be the main methods of treatment [2].

Among the many possible postoperative complications, anastomotic leakage (AL) is the most common and serious [3]. Although its incidence is gradually decreasing due to the continuous improvement of surgical methods and the gradual popularization of new technologies such as video-assisted or robot-assisted thoracic surgery, AL still occurs in 8.5–25.6% of patients after esophagectomy [4–9]. AL can not only lead to mediastinitis, peritonitis and other infections, but also to anastomotic stricture, the need for re-operation and recurrence, resulting in prolonged hospital stay and increased mortality [10–13]. Therefore, accurate prediction of the occurrence and prompt prevention of AL are essential to accelerate the recovery of patients, improve their quality of life and prolong their life survival.

Systemic inflammatory response and nutritional status are closely related to AL. Malnutrition is one of the most important systemic factors causing AL, and the occurrence and development of AL will raise strong systemic inflammatory response [14, 15]. Various inflammatory and nutritional markers, such as C-reactive protein-to-albumin ratio (CRP/ALB ratio), neutrophil to lymphocyte ratio (NLR), platelet to lymphocyte ratio (PLR), lymphocyte to monocyte ratio (LMR), prognostic nutritional index (PNI), have been confirmed as prognostic indicators of esophageal cancer [16–20]. However, the studies regarding predictive value for postoperative AL in patients with esophageal cancer were still few. Therefore, the purpose of this study was to explore the ability of various inflammatory and nutritional markers to predict postoperative AL in patients after esophagectomy.

## Methods

### Patients

This retrospective study was approved by Jinling Hospital institutional review board and informed consent requirements were waived. From October 2019 to August 2020, 281 patients with esophageal cancer underwent esophagectomy and reconstruction of the esophageal tract at the Department of Cardiothoracic Surgery, Jinling Hospital, Nanjing, China. Among them, 8 patients were excluded for insufficient data of postoperative biochemical parameters. A total of 273 patients were retrospectively evaluated and enrolled into this study.

### Data collection

The following data were extracted and analyzed. (1) Perioperative data including gender, age, body mass index (BMI), preoperative chemotherapy and/or radiotherapy history, smoking history, thoracic operation history, preoperative comorbidities (diabetes and pulmonary diseases); (2) The surgery-related data including American Society of Anesthesiologists (ASA) score, the type of surgery, the duration of operation and nutritional pathway; (3) The tumor-related data including tumor location, tumor histology, tumor differentiation and pathological stages which were classified according to the 8th edition of the TNM classification system [21]; (4) Laboratory tests data were measured on the third postoperative day (POD 3), including white blood cell, lymphocyte, monocyte, neutrocyte, red blood cell, albumin, hemoglobin, thrombocyte, C-reactive protein and glucose.

### Inflammatory and nutritional markers

The inflammatory and nutritional markers were calculated as follows: CRP/ALB ratio [CRP (mg/L) to albumin (g/L)]; NLR [neutrophil (10^9^/L) to lymphocyte (10^9^/L)]; PLR [platelet (10^9^/L) to lymphocyte (10^9^/L)]; LMR [lymphocyte (10^9^/L) to monocyte (10^9^/L)] and PNI [albumin (g/L) + 0.005 × lymphocyte count/μL].

### Definition of AL

As the endpoint of this study, AL was defined as follows: (1) The leakage of intestinal content from the anastomosis led to clinical features (including intestinal contents found in surgical incision or chest tube drains, wound infection, mediastinitis, peritonitis, pneumothorax and empyema) and/or (2) Leakage was detected by imaging examination, endoscopy, or surgical exploration [22].

### Statistical analysis

Statistical analyses were performed using SPSS (version 22.0, SPSS Inc. Chicago, IL, USA). Categorical variables were compared using χ^2^ test or Fisher’s exact test, continuous variables were compared using Student’s t-test and sequential variables were compared using Mann-Whitney U test. The discriminatory ability and optimal cut-off value was evaluated according to the receiver operating characteristic (ROC) curve analysis. Univariate logistic regression analysis was used to assess the risk factors for AL, and multivariate analysis was performed only for those factors with *p* value less than 0.05 in the univariate model. All *p* values of less than 0.05 were considered statistically significant.

## Results

### Patient characteristics

The characteristics of eligible patients with or without AL are summarized in Table 1. A total of 273 patients were included into this study, and the incidence of AL was 12.5% (34/273). The average age of all the enrolled patients was 64.4 years, with more male patients (78.4%, 214/273) than female patients (21.6%, 59/273). The occurrence of AL was significantly associated with low ASA score (U = 3205, z = − 2.452, *p* = 0.014), open operation (*p* = 0.006), long operation time (*p* <  0.001), abnormal level of postoperative albumin (*p* <  0.001), hemoglobin (Hb) (*p* = 0.015), C-reactive protein (CRP) (*p* <  0.001), C-reactive protein to albumin ratio (CRP/ALB ratio) (*p* <  0.001) and prognostic nutritional index (PNI) (*p* = 0.034). There was also a potential correlation of male patients with AL (*p* = 0.053). However, we detected no significant associations between AL and age, BMI, preoperative chemotherapy and/or radiotherapy, diabetes, smoking, thoracic operation history, pulmonary diseases, tumor location, histology, differentiation, pathological stage, nutritional pathway, white blood cell (WBC), lymphocyte, monocyte, neutrocyte, red blood cell (RBC), thrombocyte, glucose, neutrophil to lymphocyte ratio (NLR), platelet to lymphocyte ratio (PLR) and lymphocyte to monocyte ratio (LMR) (*p* > 0.1).

**Table 1:** Clinicopathological, operative and biochemical variables associated with AL

Variable	All (*n* = 273)	Non-AL (*n* = 239)	AL (*n* = 34)	*p* value
Gender				0.053
Male	214 (78.4)	183 (76.6)	31 (91.2)	
Female	59 (21.6)	56 (23.4)	3 (8.8)	
Age (years)	64.4 ± 8.8	64.3 ± 8.9	64.8 ± 8.4	0.853
BMI (kg/m^2^)	22.94 ± 3.18	22.99 ± 3.25	22.56 ± 2.67	0.491
Preoperative chemotherapy and/or radiotherapy				0.198
No	228 (83.5)	197 (82.4)	31 (91.2)	
Yes	45 (16.5)	42 (17.6)	3 (8.8)	
Diabetes				0.720
No	253 (92.7)	222 (92.9)	31 (91.2)	
Yes	20 (7.3)	17 (7.1)	3 (8.8)	
Smoking				0.302
No	135 (50.5)	121 (50.6)	14 (41.2)	
Yes	138 (49.5)	118 (49.4)	20 (58.8)	
Thoracic operation history				0.592
No	271 (99.3)	237 (99.2)	34 (100)	
Yes	2 (0.7)	2 (0.8)	0 (0)	
Pulmonary diseases				0.271
No	270 (98.9)	237 (99.2)	33 (97.1)	
Yes	3 (1.1)	2 (0.8)	1 (2.9)	
ASA score				0.014
1	187 (68.5)	157 (65.7)	30 (88.2)	
2	72 (26.4)	70 (29.3)	2 (5.9)	
3	14 (5.1)	12 (5.0)	2 (5.9)	
Tumor location				0.336
Upper	20 (7.3)	19 (7.9)	1 (2.9)	
Middle	164 (60.1)	140 (58.6)	24 (70.6)	
Lower	89 (32.6)	80 (33.5)	9 (26.5)	
Histology				0.370
SCC	256 (93.8)	224 (93.7)	32 (94.1)	
AC	2 (0.7)	2 (0.8)	0 (0)	
ASC	2 (0.7)	1 (0.4)	1 (2.9)	
Other ^a^	13 (4.8)	12 (5.0)	1 (2.9)	
Differentiation				0.883
Well	71 (26.0)	61 (25.5)	10 (29.4)	
Moderate	158 (57.9)	139 (58.2)	19 (55.9)	
Poor	44 (16.1)	39 (16.3)	5 (14.7)	
Pathological Stage				0.199
I	104 (38.1)	90 (37.6)	14 (41.2)	
II	62 (22.7)	59 (24.7)	3 (8.8)	
III	101 (37.0)	86 (36.0)	15 (44.1)	
IV	6 (2.2)	4 (1.7)	2 (5.9)	
Operation type				0.006
Open operation	137 (50.2)	128 (53.6)	9 (26.5)	
VATS	97 (35.5)	77 (32.2)	20 (58.8)	
RATS	39 (14.3)	34 (14.2)	5 (14.7)	
Duration of operation (hours)	3.96 ± 1.18	3.83 ± 1.15	4.88 ± 0.97	< 0.001
Nutritional pathway				0.217
Jejunostomy tube	31 (11.4)	25 (10.5)	6 (17.6)	
Nasojejunal tube	242 (88.6)	214 (89.5)	28 (82.4)	
Postoperative WBC (10^9^/L)	11.14 ± 3.36	11.19 ± 3.33	10.80 ± 3.62	0.671
Postoperative lymphocyte (10^9^/L)	0.69 ± 0.35	0.70 ± 0.36	0.61 ± 0.29	0.194
Postoperative monocyte (10^9^/L)	0.61 ± 0.25	0.60 ± 0.24	0.61 ± 0.32	0.871
Postoperative neutrocyte (10^9^/L)	9.77 ± 3.19	9.81 ± 3.18	9.51 ± 3.30	0.791
Postoperative RBC (10^12^/L)	3.89 ± 0.60	3.91 ± 0.58	3.75 ± 0.67	0.273
Postoperative albumin (g/L)	33.75 ± 3.69	34.11 ± 3.50	31.2 ± 0	< 0.001
Postoperative Hb (g/L)	118.5 ± 19.0	119.3 ± 19.0	112.5 ± 17.7	0.015
Postoperative platelet (10^9^/L)	179.08 ± 56.87	178.91 ± 55.37	180.29 ± 67.43	0.703
Postoperative CRP (mg/L)	71.19 ± 63.71	56.45 ± 47.70	174.82 ± 66.35	< 0.001
Postoperative glucose (mmol/L)	8.42 ± 2.94	8.50 ± 3.03	7.86 ± 2.13	0.313
Postoperative CRP/ALB ratio	2.19 ± 2.12	1.69 ± 1.50	5.74 ± 2.43	< 0.001
Postoperative NLR	18.74 ± 13.02	18.41 ± 12.64	21.04 ± 15.42	0.482
Postoperative PLR	320.31 ± 186.99	312.87 ± 176.75	372.62 ± 244.30	0.239
Postoperative LMR	1.42 ± 1.31	1.42 ± 1.32	1.36 ± 1.23	0.442
Postoperative PNI	68.29 ± 18.48	69.21 ± 18.74	61.81 ± 15.27	0.034

Data are presented as n (%) or mean ± standard deviation (SD)

*AL* anastomotic leakage, *BMI* body mass index, *ASA score* American Society of Anesthesiologists (ASA) score, *SCC* squamous cell carcinoma, *AC* adenocarcinoma, *ASC* adenosquamous carcinoma, *VATS* video-assisted thoracic surgery, *RATS* robot-assisted thoracic surgery, *WBC* white blood cell, *RBC* red blood cell, *Hb* hemoglobin, *CRP* C-reactive protein, *CRP/ALB ratio* C-reactive protein to albumin ratio, *NLR* neutrophil to lymphocyte ratio, *PLR* platelet to lymphocyte ratio, *LMR* lymphocyte to monocyte ratio, *PNI* prognostic nutritional index

^a^ Carcinoid tumors (*n* = 9), sarcomas (*n* = 2) and lymphomas (*n* = 2)

### Cut-off value of inflammatory and nutritional markers

**Fig. 1:**
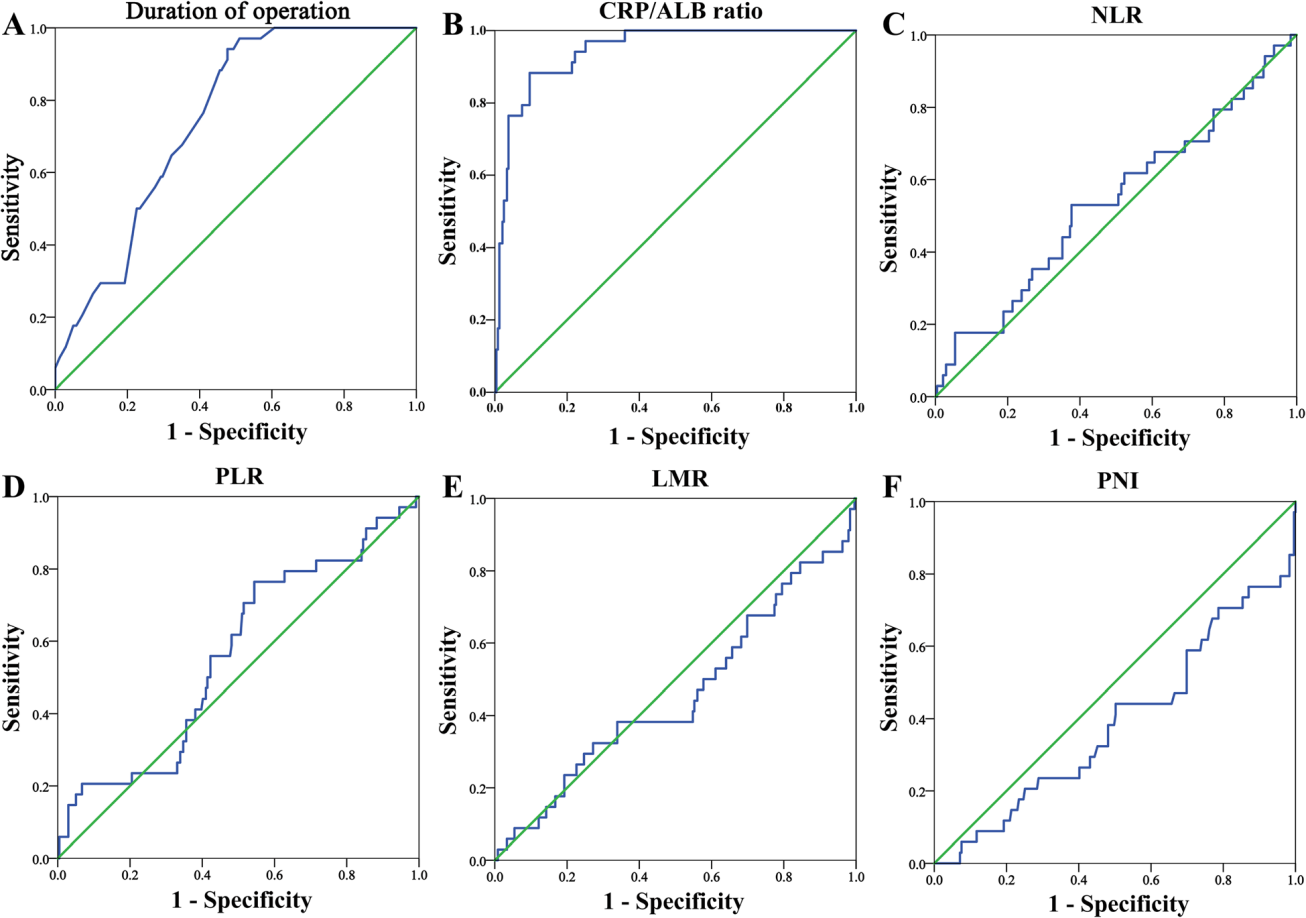
The receiver operating characteristic (ROC) curves for the Duration of operation (**a**), CRP/ALB ratio (**b**), NLR (**c**), PLR (**d**), LMR (**e**), and PNI (**f**). CRP/ALB ratio, C-reactive protein to albumin ratio; NLR, neutrophil to lymphocyte ratio; PLR, platelet to lymphocyte ratio; LMR, lymphocyte to monocyte ratio; PNI, prognostic nutritional index

The optimal cut-off value was determined according to the receiver operating characteristic (ROC) curve (Table 2 and Fig. 1). Among them, CRP/ALB ratio [AUC 0.943 (95% confidence interval (CI) = 0.911–0.976, *p* <  0.001)] and operation time [AUC 0.747 (95% CI = 0.679–0.815, *p* <  0.001)] had the greatest discrimination on AL prediction, PNI [AUC 0.612 (95% CI = 0.506–0.719, *p* = 0.034)] were also effective predictors for AL.

**Table 2:** Receiver operating characteristic analysis for each factor

Variable	Cut off	Sensitivity	Specificity	AUC(95% CI)	*p* value
Duration of operation (hours)	3.65	94.12%	52.30%	0.747 (0.679–0.815)	< 0.001
Postoperative CRP/ALB ratio	3.00	88.24%	90.38%	0.943 (0.911–0.976)	< 0.001
Postoperative NLR	18.38	52.95%	62.34%	0.537 (0.428–0.646)	0.482
Postoperative PLR	255.00	76.47%	45.61%	0.562 (0.460–0.664)	0.239
Postoperative LMR	1.04	61.76%	54.81%	0.541 (0.429–0.652)	0.442
Postoperative PNI	58.90	52.94%	69.87%	0.612 (0.506–0.719)	0.034

*AUC* area under the curve, *CI* confidence interval, *CRP/ALB ratio* C-reactive protein to albumin ratio, *NLR* neutrophil to lymphocyte ratio, *PLR* platelet to lymphocyte ratio, *LMR* lymphocyte to monocyte ratio, *PNI* prognostic nutritional index

### Risk factors for AL

C-reactive protein and albumin were excluded from the univariate and multivariate models due to their statistical correlation with CRP/ALB ratio. Lymphocyte, monocyte, neutrocyte and platelet were excluded for the same reason. Univariate analysis showed that ASA score, operation type, operation time, postoperative Hb, CRP/ALB ratio, PLR and PNI were risk factors for AL (*p* <  0.05), whereas multivariate analysis demonstrated that CRP/ALB ratio and operation time were two independent risk factors for AL, and CRP/ALB ratio (OR = 102.909, *p* <  0.001) had an advantage over operation time (OR = 9.363, *p* = 0.020; Table 3).

**Table 3:** Univariate and multivariate analysis of factors affecting AL after esophagectomy

Variable	Number (%)	Univariate analysis		Multivariate analysis	
		OR(95% CI)	*p* value	OR(95% CI)	*p* value
Age (years)				0.995(0.925–1.069)	0.882
BMI (kg/m^2^)				0.918(0.735–1.147)	0.453
ASA score			0.014		0.169
1	187(68.5)	1		1	
2	72(26.4)	0.150(0.035–0.643)		0.143(0.017–1.182)	
3	14(5.1)	0.872(0.186–4.097)		0.421(0.033–5.439)	
Operation type			0.006		0.246
Open operation	137(50.2)	1		1	
VATS	97(35.5)	3.694(1.601–8.522)		2.656(0.540–13.073)	
RATS	39(14.3)	2.092(0.658–6.650)		0.693(0.103–4.686)	
Duration of operation (hours)			0.000		0.020
<3.7	127(46.5)	1		1	
≥ 3.7	146(53.5)	17.544(4.111–74.861)		9.363(1.416–61.909)	
Postoperative WBC (10^9^/L)			0.870		0.145
<9.5	85(31.1)	1		1	
≥ 9.5	188(68.9)	0.938(0.435–2.023)		0.318(0.068–20.532)	
Postoperative RBC (10^12^/L)			0.114		0.155
<4.0	134(49.1)	1		1	
≥ 4.0	139(50.9)	0.555(0.266–1.160)		3.562(0.618–20.532)	
Postoperative Hb (g/L)			0.001		0.121
<120	130(47.6)	1		1	
≥ 120	143(52.4)	0.282(0.126–0.630)		0.246(0.042–1.446)	
Postoperative glucose (mmol/L)				0.945(0.772–1.156)	0.582
Postoperative CRP/ALB ratio			0.000		< 0.001
<3.00	220(80.6)	1		1	
≥ 3.00	53(19.4)	70.435(22.790–217.687)		102.909(22.522–470.224)	
Postoperative NLR			0.088		0.788
<18.38	165(60.4)	1		1	
≥ 18.38	108(39.6)	1.863(0.904–3.836)		1.318(0.176–9.874)	
Postoperative PLR			0.015		0.254
<255.00	117(42.9)	1		1	
≥ 255.00	156(57.1)	2.725(1.186–6.264)		2.818(0.476–16.689)	
Postoperative LMR			0.070		0.851
<1.04	129(47.3)	1		1	
≥ 1.04	144(52.7)	0.510(0.244–1.067)		0.843(0.142–5.024)	
Postoperative PNI			0.008		0.856
<58.90	90(33.0)	1		1	
≥ 58.90	183(67.0)	0.383(0.185–0.794)		1.219(0.125–11.888)	

Data are presented as n (%)

*OR* odds ratio, *CI* confidence interval, *BMI* body mass index, *ASA score* American Society of Anesthesiologists (ASA) score, *VATS* video-assisted thoracic surgery, *RATS* robot-assisted thoracic surgery, *WBC* white blood cell, *RBC* red blood cell, *Hb* hemoglobin, *CRP/ALB ratio* C-reactive protein to albumin ratio, *NLR* neutrophil to lymphocyte ratio, *PLR* platelet to lymphocyte ratio, *LMR* lymphocyte to monocyte ratio, *PNI* prognostic nutritional index

## Discussion

As the most common and serious postoperative complications after esophagectomy, AL has always been the focus of research. People have been constantly exploring the most closely related risk factors in order to accurately predict the occurrence of AL. In the present study, seven risk factors for AL including ASA score, operation type, operation time, postoperative Hb, CRP/ALB ratio, PLR and PNI were shown in univariate analysis. However, multivariate analysis identifies that only postoperative CRP/ALB ratio and operation time were two independent risk factors for AL. The overall incidence of AL in this study was 12.5%, which was similar to that reported in other clinical reports.

Lower BMI have been reported to be associated with high risk of AL by Scipione et al. [23] which may be attributed to nutritional status. A study of non-surgical factors for AL found low hemoglobin predisposes to AL as a result of abnormal hemodynamic and oxygenation [24]. In several previous studies, no relationship between ASA score and AL was found [24, 25]. In our univariate analysis, the incidence of AL was lower in patients with score 2 than in patients with score 1, though the overall condition of a score 2 was poorer. Further research is needed to confirm this result and analyze its biological mechanism. As revealed by the Society of Thoracic Surgeons [26], operation time greater than 5 h was a significant risk factor for AL. Our results showed that the optimal cut-off value was 3.7 h. Long operation time reflects difficulties during operation, leading to technical failure of anastomosis. A meta-analysis by Guo et al. [27] found no significant difference in AL between video-assisted thoracic surgery (VATS) and open surgery. In our study, univariate analysis showed that VATS had an increased risk of AL, but no significant difference were found between robot-assisted thoracic surgery (RATS) and open surgery. Consistent with our results, Lipska et al. [25] also found that male patients were more likely to develop AL, which may be related to hormonal differences affecting intestinal microcirculation. No previous study reported age and TNM stage to be associated with increased risk of AL, nor did our study. Girard et al. [28] reported diabetes and smoking increase the risk of AL, however, this association were not found in this present study.

The biological mechanism of AL has not been adequately researched. It is widely accepted that any reason cause ischemia of the alimentary canal will lead to poor healing of anastomosis. Immune and nutritional status have been demonstrated to be associated with postoperative complications and survival in previous studies [3]. Several studies have confirmed that low serum protein can affect the blood supply and oxygenation of the anastomosis, making it more prone to AL [29, 30]. Serum albumin is an indicator of nutritional status and hypoalbuminemia usually leads to a poor prognosis. Also, it can also be used as an acute phase protein to mediate inflammatory response [31]. According to Warschkow et al. [32], the serum concentration of CRP reflected the stimulation intensity of acute inflammatory reaction and increased before the development of postoperative infectious complications, and the POD 3 or 4 CRP level was widely recommended for the prediction and diagnosis of postoperative AL [33, 34].

Compared with CRP or albumin alone, CRP/ALB ratio is a preferable indicator of immune response and nutritional status [35]. And CRP/ALB ratio has been used as a prognostic factor in variety of solid cancers including esophageal cancer, lung cancer, gastric cancer, colorectal cancer and so on [36–39]. Recent reports by Yu et al. have found preoperative CRP/ALB ratio was an independent risk factor for AL in elderly colorectal cancer patients with a cut-off value of 2.44 [40]. Our study further indicated that postoperative CRP/ALB ratio was also an independent risk factor for AL after esophagectomy. According to our analysis, the optimal cut-off value of postoperative CRP/ALB ratio was 3.00.

Our study has also found that PLR and PNI were risk factors for AL. They are also effective predictors of the prognosis and complications of many solid cancers as the same with CRP/ALB ratio [18, 41], and are calculated from platelet, lymphocyte and albumin. The detection of lymphocyte number in peripheral blood can reflecting the immune level to a certain extent, and the decrease of lymphocyte number indicates low function of immune system [42]. Platelets are also closely related to the development of tumors and distant metastasis. Thrombocytosis is common among patients with malignant tumors [43]. However, the pathophysiological relationship between them and AL is still not clear, which needs further study.

### Limitation

This study has several limitations. First, this is a retrospective study from our single center, and the number of patients is not sufficient enough. Second, due to lack of laboratory data, we did not compare the predictive ability of preoperative and postoperative CRP/ALB ratio for AL. Third, the biological mechanism of CRP/ALB ratio in predicting AL was still unclear. More prospectively multicenter clinical studies were needed to explore the correlation and mechanism of CRP/ALB ratio with AL in patients after esophagectomy.

## Conclusion

Operation time and postoperative CRP/ALB ratio were two independent predictive indexes for AL. Postoperative CRP/ALB ratio greater than 3.00 indicated a high risk of AL. For patients with abnormal postoperative CRP/ALB ratio, early non-operative treatment or surgical intervention are needed to reduce the serious sequelae of AL.

## Data Availability

This study is based on data retrieved from a hospital medical record system. All personal data have been protected and secured according to current national and international laws.

## Abbreviations

AL: Anastomotic leakage
EC: Esophageal cancer
CRP/ALB ratio: C-reactive protein-to-albumin ratio
NLR: Neutrophil to lymphocyte ratio
PLR: Platelet to lymphocyte ratio
LMR: Lymphocyte to monocyte ratio
PNI: Prognostic nutritional index
BMI: Body mass index
ASA score: American Society of Anesthesiologists score
POD: Postoperative day
ROC: Receiver operating characteristic
VATS: Video-assisted thoracic surgery
RATS: Robot-assisted thoracic surgery
